# Apheresis‐Based Desensitization to Reduce Antibody Titer in ABO‐Incompatible Kidney Transplantation: A Systematic Review and Meta‐Analysis

**DOI:** 10.1155/joot/5848415

**Published:** 2026-04-29

**Authors:** Teguh Triyono, Usi Sukorini, Fuad Anshori, Noka Yogahutama

**Affiliations:** ^1^ Department of Clinical Pathology and Laboratory Medicine, Faculty of Medicine, Public Health, and Nursing, Universitas Gadjah Mada, Yogyakarta, Indonesia, ugm.ac.id; ^2^ Emergency Unit, An-Nur Surgical Hospital, Yogyakarta, Indonesia

## Abstract

**Introduction and Objectives:**

The use of apheresis‐based desensitization has enabled a safe ABO‐incompatible kidney transplantation (ABOi‐KT) by reducing ABO allo‐isoagglutinin IgG and/or IgM (anti‐A and/or anti‐B) titers. However, the rate of titer reduction and the number of apheresis sessions required for apheresis‐based desensitization remain unclear. We conducted this study to summarize the effectiveness of apheresis‐based desensitization for ABOi‐KT, as reflected in IgG and IgM titer reduction rate (TRR) and the number of apheresis sessions required.

**Materials and Methods:**

A systematic literature search was performed on PubMed, Cochrane Library, ProQuest, Scopus, ScienceDirect, and MEDLINE according to the 2020 Preferred Reporting Items for Systematic Reviews and Meta‐Analyses (PRISMA) Statement up to May 2025. Twenty‐five cohort studies and twenty‐three case series studies. Risk of bias assessment was performed using the Joanna Briggs Institute (JBI) critical appraisal tools.

**Results:**

The IgG and IgM TRR based on thirty‐one and fifteen included studies involving 1105 and 642 patients, respectively, showed a statistically significant reduction in both IgG and IgM titers with pooled TRR MD of 3.73 and 3.82 Log_2_ units, respectively (95% CI 3.23 to 4.23, *I* ^2^ 96.05%, *p* < 0.001; 95% CI 3.29 to 4.35, *I* ^2^ 95.74%, *p* < 0.001). Twenty two included studies involving 934 patients showed an average of 4.97x apheresis sessions required per patient (95% CI 4.32 to 5.61, *I* ^2^ 96.44%, *p* < 0.001). Subgroup meta‐analyses based on apheresis types, publication decade, and study type were also reported with statistically significant results.

**Conclusion:**

The reported IgG and IgM TRR and apheresis sessions required per patient in apheresis‐based desensitization for ABOi‐KT showed significant results. Similar outcomes were observed among plasmapheresis (PE), immunoadsorption (IA), and the combination of both, indicating that any of these techniques may be effective. This may reflect the effectiveness of apheresis‐based desensitization for ABOi‐KT, regardless of the apheresis technique. These findings can be a consideration in developing apheresis‐based desensitization guidelines for ABOi‐KT.

## 1. Introduction

Kidney transplantation (KT) is the gold standard treatment for patients with end‐stage kidney disease (ESKD). However, the increasing number of ESKD cases over time has led to a shortage of available kidney donors and prolonged waiting times, resulting in higher morbidity and mortality among ESKD patients. To address this issue, ABO‐incompatible kidney transplantation (ABOi‐KT) has emerged as a promising strategy to expand the donor pool and improve patient survival. Nowadays, ABOi‐KT is widely practiced worldwide. Despite its potential, ABOi‐KT carries a higher risk of antibody‐mediated rejection (AMR), which may compromise long‐term graft function [1, 2]. The major immunological barrier to ABOi‐KT is the presence of preformed IgM and IgG antibodies against A and/or B blood group antigens, with IgG being particularly associated with worse outcomes [3]. These anti‐A and/or anti‐B antibodies can bind to the corresponding antigens on donor endothelial cells, leading to complement activation, cell damage, and ultimately graft loss [4, 5].

To reduce the risk of AMR, desensitization protocols involving apheresis and immunosuppressive therapy are typically performed before transplantation to lower antibody titers to acceptable levels. Apheresis is a key component of pre‐transplant desensitization, especially in patients with high baseline ABO antibody titers. Technological advances in apheresis over the past 4 decades, such as plasma exchange (PE), immunoadsorption (IA), and double filtration plasmapheresis (DFPP), have enabled effective reduction of ABO allo‐isoagglutinin immunoglobulin G (IgG) and/or immunoglobulin M (IgM) (anti‐A and/or anti‐B) titer, often aiming for a titer of ≤ 8 on the pre‐KT period [2]. The effectiveness of apheresis in lowering ABO allo‐isoagglutinin IgG and/or IgM (anti‐A and/or anti‐B) titer is a crucial determinant of ABOi‐KT success. This effectiveness can be quantified by the titer reduction rate (TRR), calculated as the difference in Log_2_‐transformed ABO allo‐isoagglutinin IgG and/or IgM (anti‐A and/or anti‐B) titer in the baseline and pre‐KT periods. However, there is currently no standardized protocol for apheresis‐based desensitization in ABOi‐KT, and no meta‐analysis has yet quantified apheresis‐based desensitization in lowering ABO allo‐isoagglutinin IgG and/or IgM (anti‐A and/or anti‐B) titer and the amount of session required in apheresis‐based desensitization. To fill this gap, we conducted a systematic review and meta‐analysis to evaluate the effectiveness of apheresis in reducing anti‐A and/or anti‐B antibody titers in ABOi‐KT, using TRR as the primary outcome measure and the average apheresis session required in desensitization as the secondary outcome.

## 2. Material and Methods

### 2.1. Literature Search Strategies and Inclusion Criteria

This systematic review and meta‐analysis was registered in the PROSPERO database (CRD420251066548). This study was conducted in accordance with the 2020 Preferred Reporting Items for Systematic Reviews and Meta‐Analyses (PRISMA) Statement [6]. A systematic literature search was performed in six databases: PubMed, Cochrane Central Register of Controlled Trials (CENTRAL), Scopus, ProQuest, ScienceDirect, and MEDLINE up to May 2025 by three reviewers (T.T., U.S., and N.Y.). The literature search was based on Medical Subject Headings (MeSH) terms and relevant keywords, as shown in Supporting Table 1. The inclusion criteria for this review were defined using the PEO framework:

 P: patients undergoing ABOi‐KT who received apheresis‐based desensitization;
 E: apheresis‐based desensitization for ABOi‐KT;
 O: change of ABO allo‐isoagglutinin IgG and/or IgM (anti‐A and/or anti‐B) titer from baseline until pre‐KT periods calculated as TRR (primary outcome) and the average number of apheresis session required per patient for desensitization (secondary outcome).

Additional inclusion criteria were as follows: (1) studies mentioning ABO allo‐isoagglutinin IgG and/or IgM (anti‐A and/or anti‐B) titer as an individual participant data (IPD) or median with range and/or interquartile range (IQR) data; (2) human studies; and (3) studies published in English language. Exclusion criteria were as follows: (1) review, case report, letters, or editorial studies; (2) abstract‐only or conference studies; (3) nonhuman studies; and (4) studies not published in English language. No randomized controlled trial (RCT) studies meeting the inclusion criteria were found during the literature search period. However, to reduce small‐sample bias, we also excluded case review studies that have subjects lower than 10. Any duplicates considered in literature search were deleted using automated tools of Rayyan.ai [7].

### 2.2. Data Extraction and Study Quality

Data of author, study origin, study period, study type, patient age and gender, and type of apheresis techniques were obtained. Data required for meta‐analysis included the number of ABO allo‐isoagglutinin IgG and/or IgM (anti‐A and/or anti‐B) titer at baseline period, the number of ABO allo‐isoagglutinin IgG and/or IgM (anti‐A and/or anti‐B) titer at pre‐KT period, the amount of apheresis session required for desensitization, and the average amount of apheresis session required on each patient for desensitization. Values obtained from included studies were used to define outcomes in this study as follows:

1. IgG TRR: the reduction of ABO allo‐isoagglutinin IgG (anti‐A and/or anti‐B) titer from baseline to pre‐KT period after desensitization in Log_2_ calculation;
2. IgM TRR: the reduction of ABO allo‐isoagglutinin IgM (anti‐A and/or anti‐B) titer from baseline to pre‐KT period after desensitization in Log_2_ calculation;
3. Apheresis session per patient: the average number of apheresis sessions required per patient for desensitization.

All of the data obtained from included studies were calculated, extracted, and cross‐checked using Microsoft Excel version 15.20 by three reviewers (U.S., F.A., and N.Y.).

### 2.3. Risk of Bias Assessment

Risk of bias assessment was performed by two reviewers (T.T. and N.Y.) using Joanna Briggs Institute (JBI)’s critical appraisal tools for the cohort and case series studies with 11 and 10 questions, respectively [8, 9]. All of the questions from JBI’s critical appraisal tools were answered based on choices of “yes, no, and unclear”. Any different answer on each question was discussed between two reviewers (T.T. and N.Y.) and decided by the first author (T.T.). A percentage of “yes” answers was calculated, and an overall risk of bias category was assigned by the first author (T.T.) based on the percentage score: 0–< 40% was categorized as “high”; 40–< 70% as “moderate”; and 70%–100% as “low”. Studies categorized as moderate and low according to overall risk of bias category were included.

### 2.4. Statistical Analysis

Meta‐analyses were performed when two or more studies reported the same outcomes. For continuous data presented as medians with ranges and/or IQR, we used formulas described by Wan et al. and Luo et al., as recommended by the Cochrane Handbook for Systematic Reviews of Interventions [10–12], to convert these values into means and standard deviations (SD). To combine continuous data between groups, we applied the formula recommended by the Cochrane Handbook for Systematic Reviews of Interventions, assuming a correlation coefficient (*r*) of 0.5 [12]. Mean difference (MD) with a random‐effects model using restricted maximum likelihood (REML) estimation was used to analyze continuous data. ABO allo‐isoagglutinin IgG and/or IgM (anti‐A and/or anti B) titer with semiquantitative data was transformed into Log_2_ calculation for meta‐analyses. Semiquantitative titer values of 0 or < 1 were converted to 1 for Log_2_ calculation. A 1 step reduction on the Log_2_ scale reflects a 2‐fold decrease in semiquantitative antibody titer. All MD results were presented with 95% confidence interval (CI) with *p*‐value (*p*) of less than 0.05 considered as statistically significant. Heterogeneity was described in *I* ^2^ value and classified as low (< 40%), moderate (30%–60%), substantial (50%–90%), and considerable (75%–100%) according to the Cochrane Handbook for Systematic Reviews of Interventions [10]. Considering the variability of our included studies, the random‐effects model was used in all of the meta‐analysis calculations. All of the statistical analyses were generated and presented through forest plots. Egger’s test and Begg’s test were performed with (*p*) of less than 0.05 indicating a potential publication bias. Sensitivity analysis was also performed with generated forest plots using leave‐one‐out (LOO) method and funnel plots. Univariable metaregression analysis of publication year presented as bubble plot and multivariable metaregression analysis of type of apheresis, publication decade, and study type were performed with a random‐effects model using REML estimation to assess potential sources of heterogeneity on each outcome. All meta‐analyses and metaregression analyses of the included studies, Egger’s test, Begg’s test, and sensitivity analyses were performed using STATA/MP version 17 software (StataCorp, USA) by two reviewers (T.T. and N.Y.).

## 3. Results

### 3.1. Literature Search and Study Characteristics of Included Studies

**FIGURE 1:**
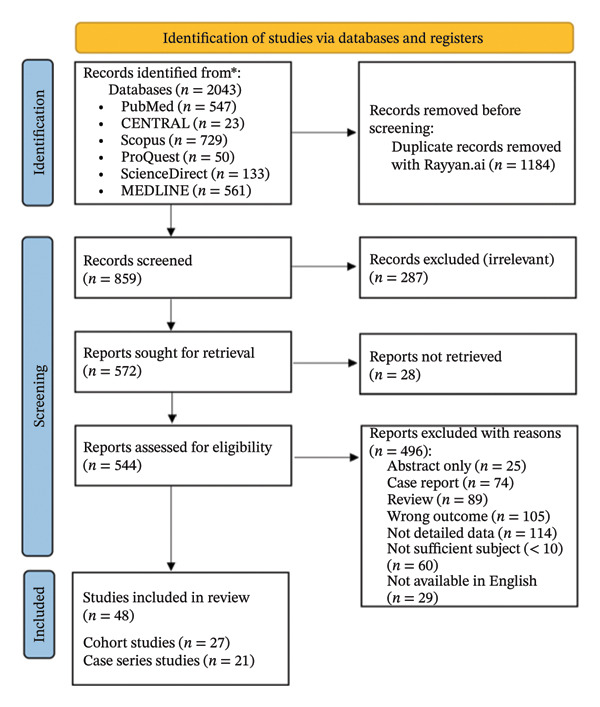
PRISMA flowchart.

The literature search was performed according to the 2020 PRISMA Statement flow chart and retrieved 2043 studies from 5 databases, as shown in Figure 1. A total of 1184 duplicates were removed using automated tools of Rayyan.ai, resulting in 859 studies that were screened based on the titles and abstracts. Of these, 287 irrelevant studies were excluded. Twenty‐eight studies could not be retrieved, and the full text of 544 studies were screened for eligibility. A total of 48 [13–35], [36–60] out of 544 studies, met the inclusion criteria, consisting of 25 cohort studies [21, 23–25, 28, 30, 33, 34, 36–39, 42, 45, 46, 48, 49, 51, 52, 54–56, 58–60] and 23 case series studies [13–20, 22, 26, 27, 29, 31, 32, 35, 40, 41, 43, 44, 47, 50, 53, 57]. The complete baseline characteristics of the included studies are presented in Supporting Table 2. A total of 1756 patients were included in this study. A summary of baseline characteristics from the included studies is presented in Table 1, and the classification of included studies based on study type and publication year is presented in Table 2. The average age from 1570 patients was 45.38 ± 13.11 (mean ± SD). In this study, male patients were dominant compared to female patients among 1566 patients (1025 vs 541 patients), respectively. The average number of apheresis sessions required per patient for desensitization protocol was 5.05 ± 5.17 (mean ± SD) among 870 patients. Most of the patients (187 patients) were categorized as ABO blood group O and received kidney donors from ABO blood group B. The summary of meta‐analysis and multivariable metaregression results are presented in Tables 3 and 4. Several studies were not included together in the same meta‐analysis outcome calculations due to the possibility of sharing the same cohorts.

**TABLE 1:** Summary of the baseline characteristics of the included studies.

No. of studies	48
Region	
Europe	15
Asia	27
America	6
Patients	
No. of patients	1756
Age	
No. of patients (known/unknown)	1570/186
Mean ± SD	45.38 ± 13.11
Gender	
No. of patients (known/unknown)	1566/190
M/F	1025/541
Apheresis	
No. of studies of apheresis type (PE/IA/combination of PE and IA)	26/10/12
Apheresis session	
No. of patients (known/unknown)	392/1364
No. of apheresis sessions	2402
Apheresis session/patient	
No. of patients (known/unknown)	870/886
Mean ± SD	5.05 ± 5.17
ABO donor/recipient pair	
No. of patients (known/unknown)	1117/639
A/AB	7
A/B	150
A/O	236
B/AB	11
B/A	167
B/O	187
AB/A	87
AB/B	127
AB/O	20
O/AB	6
O/A	75
O/B	44

*Note:* No., amount; IA, immunoadsorption; M, male; F, female.

Abbreviations: PE, plasma exchange; SD, standard deviation.

**TABLE 2:** Summary of the baseline characteristics of the included studies, classified by study type and publication year.

**Cohort studies**							
**No. of studies based on publication year**		**Total (*n* = 25)**	**2000–2005 (*n* = 0)**	**2006–2010 (*n* = 5)**	**2011–2015 (*n* = 4)**	**2016–2020 (*n* = 11)**	**2021–2025 (*n* = 5)**

Patient characteristics							
No. of patients	*n*	1427	—	155	310	713	249
No. of studies with known age	*n*	20	—	3	4	8	5
Known age	No. of patients (mean ± SD)	1292 (45.81 ± 12.48)	—	96 (46.52 ± 12.6)	310 (44.72 ± 12.4)	637 (45.51 ± 11.99)	249 (47.66 ± 13.59)
No. of studies with known gender	n	22	—	4	4	9	5
Known gender	No. of patients (M/F)	1334 (882/452)	—	109 (54/55)	310 (208/102)	666 (438/228)	249 (182/67)
Apheresis							
No. of studies of apheresis type (PE/IA/combination of PE and IA)	*n*	18/2/5	—	5/0/0	3/1/0	8/0/3	2/1/2
No. of studies with known no. of apheresis sessions	*n*	5	—	0	0	2	3
Known no. of apheresis session	No. of patients (no. of apheresis sessions)	120 (555)	—	0 (0)	0 (0)	46 (209)	74 (346)
Known apheresis session/patient	No. of patients (mean ± SD)	1059 (4.26 ± 1.82)	—	106 (7.02 ± 5.03)	310 (4.93 ± 3.04)	536 (4.12 ± 1.78)	107 (4.84 ± 1.89)

**Case series studies**							
**No. of studies based on publication year**		**Total (*n* = 23)**	**2000–2005 (*n* = 3)**	**2006–2010 (*n* = 8)**	**2011–2015 (*n* = 4)**	**2016–2020 (*n* = 7)**	**2021–2025 (*n* = 1)**

Patient characteristics							
No. of patients	*n*	329	36	118	57	108	10
No. of studies with known age	*n*	20	3	6	4	6	1
Known age	No. of patients (mean ± SD)	278 (43.38 ± 15.59)	36 (18.14 ± 13.87)	86 (45.74 ± 11.32)	57 (47.88 ± 11.47)	89 (48.02 ± 12.66)	10 (47.1 ± 12.97)
No. of studies with known gender	*n*	16	3	4	4	4	1
Known gender	No. of patients (M/F)	232 (143/89)	36 (24/12)	60 (41/19)	57 (31/26)	69 (42/27)	10 (5/5)
Apheresis							
No. of studies of apheresis type (PE/IA/combination of PE and IA)	*n*	8/8/7	0/1/2	1/6/1	3/0/1	3/1/3	1/0/0
No. of studies with known no. of apheresis sessions	*n*	19	1	8	4	5	1
Known no. of apheresis sessions	No. of patients (no. of apheresis sessions)	272 (1847)	10 (49)	118 (610)	57 (365)	77 (769)	10 (54)
Known apheresis session/patient	No. of patients (mean ± SD)	272 (8.39 ± 10.61)	10 (4.9 ± 1.91)	118 (5.17 ± 2.28)	57 (6.4 ± 3.7)	77 (9.98 ± 14.71)	10 (5.4 ± 1.65)

*Note:* No., amount; *n*, number; IA, immunoadsorption.

Abbreviations: PE, plasma exchange; SD, standard deviation.

**TABLE 3:** Summary of meta‐analysis results for the included studies and publication bias analysis using Egger’s and Begg’s tests.

Meta‐analysis	Subgroup	No. of included studies	References	Patients (n)	MD (95% CI)	*I* ^2^ (%)	*p*	Egger’s test		Begg’s test	
								*t*	*p*	z	*p*
IgG TRR	Overall	31	[14, 17–20, 22, 24, 27, 30–33, 35–39, 42, 44–48, 51, 53–56, 58–60]	1105	3.73 (3.23 to 4.23)	96.05	^<^ 0.001^∗^	0.16	0.87	−1.17	0.25
	*Apheresis type*										
	PE	16	[22, 24, 30–33, 37–39, 44–46, 53, 54, 56, 58]	742	3.63 (2.94 to 4.32)	96.45	^<^ 0.001^∗^	−0.4	0.69	−0.77	0.5
	IA	6	[17–19, 27, 36, 59]	102	4.11 (3.31 to 4.92)	84.22	^<^ 0.001^∗^	−0.53	0.62	−0.57	0.85
	PE ± IA	9	[14, 20, 35, 42, 47, 48, 51, 55, 60]	261	3.67 (2.54 to 4.79)	97.24	^<^ 0.001^∗^	1.1	0.31	−0.1	1
	*Publication decade*										
	2000–2009	7	[14, 17–20, 22, 24]	122	3.65 (2.76 to 4.54)	91.16	^<^ 0.001^∗^	−1.16	0.3	−0.77	0.64
	2010–2019	17	[27, 30–33, 35–39, 42, 44–48, 51]	604	3.71 (3.08 to 4.35)	95.3	^<^ 0.001^∗^	0.79	0.44	−0.95	0.39
	2020–2024	7	[53–56, 58–60]	331	3.85 (2.46 to 5.25)	98.32	^<^ 0.001^∗^	0.3	0.78	−0.6	0.76
	*Study type*										
	Cohort	18	[24, 30, 33, 36–39, 42, 45, 46, 48, 51, 54–56, 58–60]	892	3.89 (3.15 to 4.64)	97.8	^<^ 0.001^∗^	1.72	0.1	0.23	0.82
	CS	13	[14, 17–20, 22, 27, 31, 32, 35, 44, 47, 53]	213	3.51 (2.97 to 4.06)	83.86	^<^ 0.001^∗^	−0.84	0.42	−0.98	0.39

IgM TRR	Overall	15	[14, 16, 17, 25, 26, 30–32, 36, 42, 49, 53, 54, 59, 60]	642	3.82 (3.29 to 4.34)	95.74	^<^ 0.001^∗^	−0.84	0.41	−0.3	0.84
	*Apheresis type*										
	PE	7	[25, 30–32, 49, 53, 54]	442	4.12 (3.27 to 4.96)	97.12	^<^ 0.001^∗^	−1.52	0.19	−0.76	0.65
	IA	5	[16, 17, 26, 36, 59]	80	3.54 (2.91 to 4.18)	80.67	^<^ 0.001^∗^	0.1	0.92	−0.73	0.81
	PE ± IA	3	[14, 42, 60]	120	3.58 (2.1 to 5.07)	96.8	^<^ 0.001^∗^	8.57	0.07	1.04	0.3
	*Publication decade*										
	2000–2009	5	[14, 16, 17, 25, 26]	74	3.83 (3 to 4.65)	86.21	^<^ 0.001^∗^	−0.56	0.61	−0.73	0.81
	2010–2019	6	[30–32, 36, 42, 49]	331	4 (3.32 to 4.69)	93.93	^<^ 0.001^∗^	−0.63	0.56	0.38	0.71
	2020–2024	4	[53, 54, 59, 60]	237	3.54 (2.03 to 5.04)	98.4	^<^ 0.001^∗^	−0.55	0.64	−1.02	0.73
	*Study type*										
	Cohort	8	[25, 30, 36, 42, 49, 54, 59, 60]	454	3.8 (2.95 to 4.64)	98.05	^<^ 0.001^∗^	−1.1	0.31	−1	0.45
	CS	7	[14, 16, 17, 26, 31, 32, 53]	114	3.83 (3.21 to 4.45)	81.88	^<^ 0.001^∗^	−0.02	0.98	−0.46	0.88

Apheresis session/patient	Overall	22	[17–20, 27, 30–33, 35–39, 45, 48, 49, 53, 55, 56, 58, 59]	934	4.97 (4.32 to 5.61)	96.44	^<^ 0.001^∗^	4.3	< 0.001^∗^	2.3	0.04^∗^
	*Apheresis type*										
	PE	13	[30–33, 37–39, 45, 48, 49, 53, 56, 58]	760	4.32 (3.83 to 4.8)	92.36	^<^ 0.001^∗^	0.57	0.58	0.06	0.95
	IA	6	[17–19, 27, 36, 59]	118	6.36 (3.7 to 9.02)	97.8	^<^ 0.001^∗^	5.34	0.006^∗^	1.88	0.06
	PE ± IA	3	[20, 35, 55]	56	6.38 (4.85 to 7.91)	82.61	^<^ 0.001^∗^	0.7	0.61	0	1
	*Publication decade*										
	2000–2009	4	[17–20]	67	5.34 (4.04 to 6.65)	91.14	^<^ 0.001^∗^	1.21	0.35	1.02	0.31
	2010–2019	13	[27, 30–33, 35–39, 45, 48, 49]	714	4.63 (4.01 to 5.24)	94.2	^<^ 0.001^∗^	2.45	0.03^∗^	0.73	0.46
	2020–2024	5	[53, 55, 56, 58, 59]	153	5.83 (2.71 to 8.94)	98.9	^<^ 0.001^∗^	4.77	0.02^∗^	1.71	0.09
	*Study type*										
	Cohort	13	[30, 33, 36–39, 45, 48, 49, 55, 56, 58, 59]	783	5.2 (4.12 to 6.29)	98.05	^<^ 0.001^∗^	3.79	0.003^∗^	1.22	0.22
	CS	9	[17–20, 27, 31, 32, 35, 53]	151	4.81 (3.89 to 5.72)	92.29	^<^ 0.001^∗^	2.51	0.04^∗^	1.47	0.14

Apheresis session/patient by baseline IgG titer (≤ 64 vs > 64) to achieve pre‐KT IgG titer ≤ 16	Overall	10	[17, 19, 27, 29, 37, 41, 53, 55, 56, 59]	183	−1.04 (−1.38 to −0.7)	0	^<^ 0.001^∗^	−2.49	0.04	−1.97	0.07
	*Apheresis type*										
	PE	4	[29, 37, 53, 56]	101	−1.22 (−1.87 to −0.57)	27.67	^<^ 0.001^∗^	−1.03	0.41	−0.34	1
	IA	4	[17, 19, 27, 59]	58	−1.19 (−2.4 to 0.03)	75.36	0.05	−2.16	0.16	−0.34	1
	PE ± IA	2	[41, 55]	34	−1.04 (−1.74 to −0.33)	0	0.04^∗^	—	—	—	—
	*Publication decade*										
	2000–2009	2	[17, 19]	35	−0.71 (−1.41 to −0.01)	0	0.05	—	—	—	—
	2010–2019	4	[27, 29, 37, 41]	50	−1.89 (−2.56 to −1.22)	0	^<^ 0.001^∗^	−1.63	0.24	−1.02	0.73
	2020–2024	4	[53, 55, 56, 59]	98	−0.78 (−1.25 to −0.3)	0	0.001^∗^	−0.33	0.77	−1.02	0.73
	*Study type*										
	Cohort	4	[37, 55, 56, 59]	88	−0.94 (−1.39 to −0.48)	0	^<^ 0.001^∗^	−1.18	0.36	−1.7	0.31
	CS	6	[17, 19, 27, 29, 41, 53]	95	−1.23 (−1.83 to −0.63)	25.18	^<^ 0.001^∗^	−2.2	0.09	−1.88	0.13

*Note:* No., amount; IA, immunoadsorption; *I* ^2^, heterogeneity; *p*, *p*‐value.

Abbreviations: 95% CI, 95% confidence interval; CS, case series; MD, mean difference; PE, plasma exchange.

^∗^Statistically significant.

**TABLE 4:** Summary of multivariable metaregression analyses of the included studies.

Metaregression	Covariate	*β* (95% CI)	SE	*p*
IgG TRR	*Apheresis type*			
	PE vs IA	0.69 (−0.85–2.23)	0.78	0.38
	PE vs PE ± IA	0.12 (−1.12–1.39)	0.63	0.84
	*Publication decade*			
	2000–2009 vs 2010–2019	0.03 (−1.5–1.57)	0.78	0.96
	2000–2009 vs 2020–2024	0.05 (−1.79–1.89)	0.94	0.96
	*Study type*			
	Cohort vs CS	−0.53 (−1.81 to 0.75)	0.65	0.42

IgM TRR	*Apheresis type*			
	PE vs IA	−0.66 (−2.21 to 0.89)	−0.79	0.4
	PE vs PE ± IA	−0.55 (−2.22 to 1.12)	0.85	0.52
	*Publication decade*			
	2000–2009 vs 2010–2019	−0.16 (−1.95 to 1.61)	0.91	0.86
	2000–2009 vs 2020–2024	−0.52 (−2.39 to 1.35)	0.95	0.58
	*Study type*			
	Cohort vs CS	−0.06 (−1.5 to 1.37)	0.73	0.93

Apheresis session/patient	*Apheresis type*			
	PE vs IA	2.41 (0.35 to 4.46)	1.05	0.02^∗^
	PE vs PE ± IA	2.72 (0.53 to 4.9)	1.11	0.01^∗^
	*Publication decade*			
	2000–2009 vs 2010–2019	0.59 (−1.79–2.97)	1.21	0.63
	2000–2009 vs 2020–2024	0.61 (−2–3.23)	1.33	0.64
	*Study type*			
	Cohort vs CS	−1.16 (−2.8 to 0.48)	0.84	0.17

Apheresis session/patient by baseline IgG titer (≤ 64 vs > 64) to achieve pre‐KT IgG titer ≤ 16	*Apheresis type*			
	PE vs IA	−0.34 (−1.44 to 0.75)	−0.34	0.54
	PE vs PE ± IA	0.06 (−0.82–0.95)	0.45	0.89
	*Publication decade*			
	2000–2009 vs 2010–2019	−1.56 (−2.96 to −0.16)	0.71	0.03^∗^
	2000–2009 vs 2020–2024	−0.49 (−2 to 1.02)	0.77	0.52
	*Study type*			
	Cohort vs CS	−0.14 (−1.17 to 0.88)	0.52	0.78

*Note:* IA, immunoadsorption; *p*, *p*‐value; *β*, regression coefficient.

Abbreviations: 95% CI, 95% confidence interval; CS, case series; PE, plasma exchange; SE, standard of error.

^∗^Statistically significant.

### 3.2. IgG and IgM TRR

**FIGURE 2:**
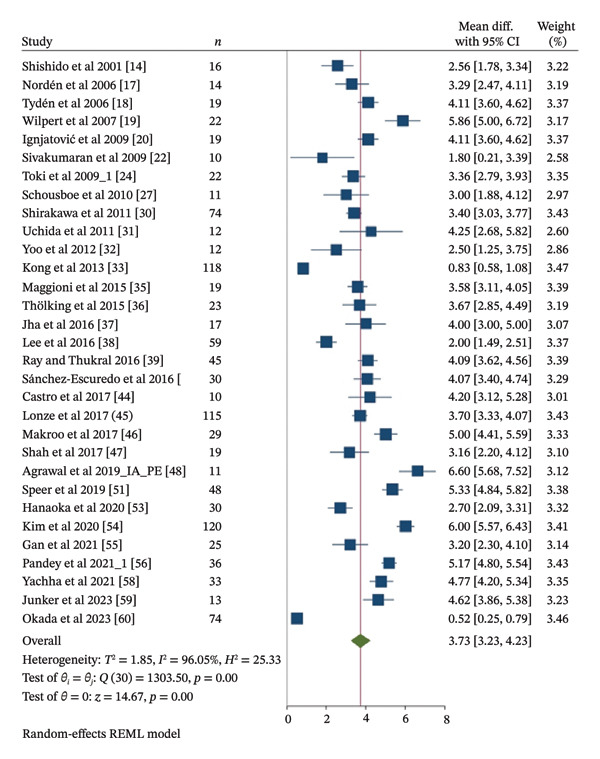
Meta‐analysis forest plot of IgG TRR.

**FIGURE 3:**
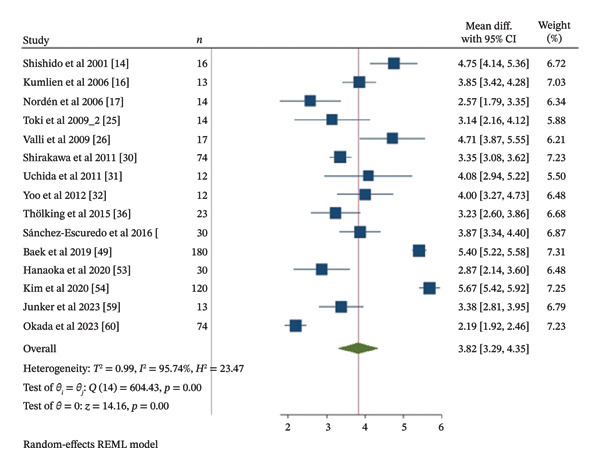
Meta‐analysis forest plot of IgM TRR.

Meta‐analysis of IgG and IgM TRR was conducted using thirty one included studies [14, 17–20, 22, 24, 27, 30–33, 35–39, 42, 44–48, 51, 53–56, 58–60] and fifteen included studies [14, 16, 17, 25, 26, 30–32, 36, 42, 49, 53, 54, 59, 60], involving 1105 and 642 patients, respectively. Apheresis was associated with a statistically significant reduction in both IgG and IgM titers, with TRR MD of 3.73 and 3.82 Log_2_ units, respectively (95% CI 3.23 to 4.23, *I* ^2^ 96.05%, *p* < 0.001; 95% CI 3.29 to 4.34, *I* ^2^ 95.74%, *p* < 0.001), as shown in Figures 2 and 3. This corresponds to approximately a 13.27‐fold reduction in IgG titers and a 14.12‐fold reduction in IgM titers.

As presented in Table 3, subgroup meta‐analyses of PE, based on sixteen included studies [22, 24, 30–33, 37–39, 44–46, 53, 54, 56, 58] for IgG TRR with 742 patients and seven included studies [25, 30–32, 49, 53, 54] for IgM TRR with 442 patients, also showed statistically significant reductions. The TRR MD for IgG and IgM titers were 3.63 and 4.12 Log_2_ units, respectively (95% CI 2.94 to 4.32, *I* ^2^ 96.45%, *p* < 0.001; 95% CI 3.27 to 4.96, *I* ^2^ 97.12%, *p* < 0.001). This corresponds to approximately a 12.38‐fold reduction in IgG titers and a 17.39‐fold reduction in IgM titers. Subgroup meta‐analyses of IA, based on six included studies [17–19, 27, 36, 59] for IgG TRR with 102 patients and five included studies [16, 17, 26, 36, 59] for IgM TRR with 80 patients, demonstrated statistically significant reductions in both IgG and IgM titers. The TRR MD for IgG and IgM titers were 4.11 and 3.54 Log_2_ units, respectively (95% CI 3.31 to 4.92, *I* ^2^ 84.22%, *p* < 0.001; 95% CI 2.91 to 4.18, *I* ^2^ 80.67%, *p* < 0.001). This translates to approximately a 17.27‐fold reduction in IgG titers and a 11.63‐fold reduction in IgM titers. The subgroup meta‐analyses of PE and IA combination, based on nine included studies [14, 20, 35, 42, 47, 48, 51, 55, 60] for IgG TRR involving 261 patients and three included studies [14, 42, 60] for IgM TRR involving 120 patients, also revealed statistically significant reductions. The TRR MD for IgG and IgM titers were 3.67 and 3.58 Log_2_ units, respectively (95% CI 2.54 to 4.79, *I* ^2^ 97.24%, *p* < 0.001; 95% CI 2.10 to 5.07, *I* ^2^ 96.8%, *p* < 0.001), corresponds to approximately 12.73‐fold and 11.96‐fold reductions in IgG and IgM titers, respectively.

The publication decade subgroup meta‐analyses of IgG and IgM TRR showed statistically significant results in all year intervals. Between 2000 and 2009, subgroup meta‐analyses of IgG and IgM TRR were conducted based on seven included studies [14, 17–20, 22, 24] with 122 patients and five included studies [14, 16, 17, 25, 26] with 74 patients showing a TRR MD of 3.65 Log_2_ units (12.55‐fold reduction) for IgG titers and 3.83 Log_2_ units (14.22‐fold reduction) for IgM titers, respectively (95% CI 2.76 to 4.54, *I* ^2^ 91.16%, *p* < 0.001; 95% CI 3 to 4.65, *I* ^2^ 86.21%, *p* < 0.001). Seventeen included studies [27, 30–33, 35–39, 42, 44–48, 51] with 604 patients and six included studies [30–32, 36, 42, 49] with 331 patients published between 2010 and 2019 were subgrouped for IgG and IgM TRR subgroup meta‐analyses. A TRR MD of 3.71 Log_2_ units (13.09‐fold reduction) for IgG titers and 4 Log_2_ units (16‐fold reduction) for IgM titers, respectively (95% CI 3.08 to 4.35, *I* ^2^ 95.3%, *p* < 0.001; 95% CI 3.32 to 4.69, *I* ^2^ 93.93%, *p* < 0.001). Subgroup meta‐analyses of IgG and IgM TRR between 2020 and 2024 were conducted using seven included studies [53–56, 58–60] involving 331 patients and four included studies [53, 54, 59, 60] involving 237 patients, respectively. The MD for IgG and IgM TRR were 3.85 Log_2_ units (95% CI 2.46 to 5.25, *I* ^2^ 98.32%, *p* < 0.001) and 3.54 Log_2_ units (95% CI 2.03 to 5.04, *I* ^2^ 98.4%, *p* < 0.001). This can be translated to approximately a 14.42‐fold reduction in IgG titers and a 11.63‐fold reduction in IgM titers.

Subgroup meta‐analyses were performed based on study type, and studies of IgG and IgM TRR were performed with eighteen included studies [24, 30, 33, 36–39, 42, 45, 46, 48, 51, 54–56, 58–60] involving 892 patients and thirteen included studies [14, 17–20, 22, 27, 31, 32, 35, 44, 47, 53] involving 213 patients for cohort and case series type of studies of IgG TRR and eight included studies [25, 30, 36, 42, 49, 54, 59, 60] involving 454 patients and seven included studies [14, 16, 17, 26, 31, 32, 53] involving 114 patients for cohort and case series type of studies of IgM TRR. The results showed an MD of 3.89 Log_2_ units (14.83‐fold reduction) for IgG and 3.8 Log_2_ units (13.93‐fold reduction) for IgM TRR of cohort studies (95% CI 3.15 to 4.64, *I* ^2^ 97.8%, *p* < 0.001; 95% CI 2.95 to 4.64, *I* ^2^ 98.05%, *p* < 0.001, respectively). The MD for IgG and IgM of case series studies were 3.51 Log_2_ units (95% CI 2.97 to 4.06, *I* ^2^ 83.86%, *p* < 0.001) and 3.83 Log_2_ units (95% CI 3.21 to 4.45, *I* ^2^ 81.88%, *p* < 0.001) that can be translated into 11.39‐fold reduction of IgG titers and 14.22‐fold reduction of IgM titers.

These meta‐analyses may indicate that apheresis type, publication decade, and study type may result in similar reductions in IgG and IgM titers with statistically significant results. However, all of the meta‐analyses resulted in a considerable heterogeneity, with *I* ^2^ values ranging from 80.67% to 98.32%. Multivariable metaregression analyses were performed to assess the potential sources of heterogeneity, as shown in Table 4. However, multivariable metaregression analysis results showed that the type of apheresis, publication decade, and study type had no statistically significant associations between those factors with IgG and IgM TRR. A univariable metaregression analysis also showed no statistically significant associations between publication year and either IgG or IgM TRR as presented in Figure 4.

FIGURE 4Univariable metaregression analysis plots of (a) IgG TRR and (b) IgM TRR with publication year.

**Figure:**
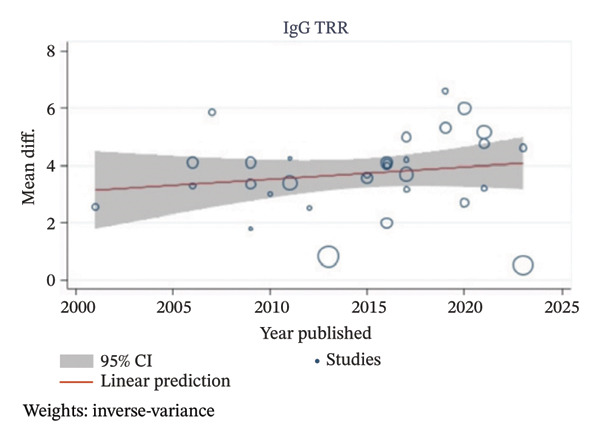
(a)

**Figure:**
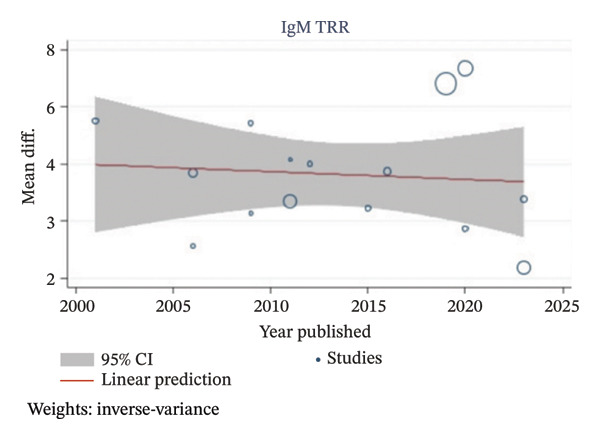
(b)

### 3.3. Apheresis Session per Patient

**FIGURE 5:**
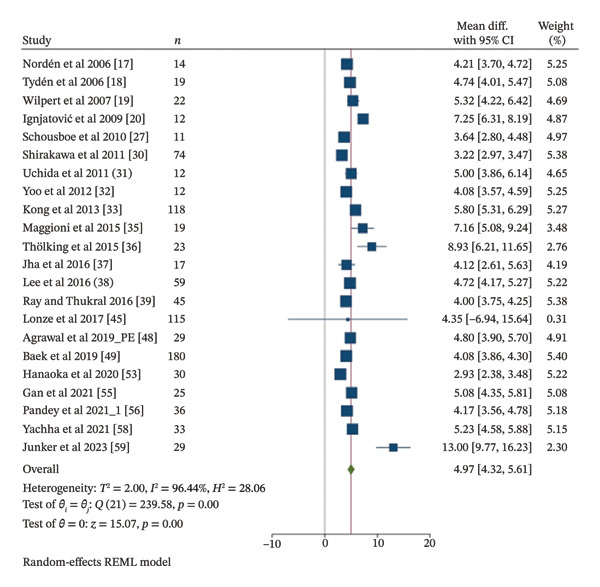
Meta‐analysis forest plot of apheresis session/patient.

A total of twenty two included studies [17–20, 22, 27, 30–33, 35–39, 48, 49, 53, 55, 56, 58, 59], involving 934 patients, reported the average number of apheresis sessions required per patient. The MD was 4.95x sessions per patient (95% CI 4.35 to 5.55, *I* ^2^ 98.23%, *p* = 0.02), as shown in Figure 5. Based on thirteen included studies [30–33, 37–39, 45, 48, 49, 53, 56, 58] involving 760 patients, subgroup meta‐analysis of PE showed a statistically significant MD of 4.32x sessions per patient (95% CI 3.83 to 4.8, *I* ^2^ 92.36%, *p* < 0.001). Subgroup meta‐analysis of IA with six included studies [17–19, 27, 36, 59] involving 118 patients and subgroup meta‐analysis of PE and IA combination with three included studies [20, 35, 55] involving 56 patients showed a statistically significant MD of 6.36x and 6.38x sessions per patient, respectively (95% CI 3.7 to 9.02, *I* ^2^ 97.8%, *p* < 0.001; 95% CI 4.85 to 7.91, *I* ^2^ 82.61%, *p* < 0.001). Subgroup meta‐analyses based on publication decade (2000–2009, 2010–2019, and 2020–2024) involving four [17–20], thirteen [27, 30–33, 35–39, 45, 48, 49], and five [53, 55, 56, 58, 59] included studies involving 67, 714, and 153 patients showed a statistically significant MD of 5.34x, 4.63x, and 5.83x sessions per patient, respectively (95% CI 4.04 to 6.65, *I* ^2^ 91.14%, *p* < 0.001; 95% CI 4.01 to 5.24, *I* ^2^ 94.2%, *p* < 0.001; 95% CI 2.71 to 8.94, *I* ^2^ 98.9%, *p* < 0.001). Subgroup meta‐analyses based on study type of case series studies with nine included studies [17–20, 27, 31, 32, 35, 53] involving 151 patients and cohort studies with thirteen included studies [30, 33, 36–39, 45, 48, 49, 55, 56, 58, 59] involving 783 patients showed a statistically significant MD of 4.81x and 5.2x sessions per patient, respectively (95% CI 3.89 to 5.72, *I* ^2^ 92.29%, *p* < 0.001; 95% CI 4.12 to 6.29, *I* ^2^ 98.05%, *p* < 0.001). The study by Castro et al. [44] was excluded from this meta‐analysis due to its potential to significantly alter the results with an unusually high number of PE sessions per patient (> 20). Nevertheless, considerable heterogeneity was observed across the meta‐analyses, with *I* ^2^ values ranging from 82.61% to 98.9%.

**FIGURE 6:**
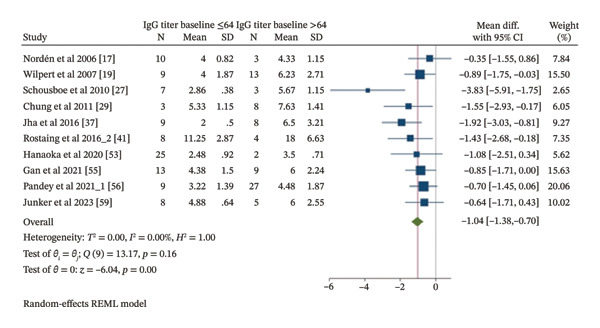
Meta‐analysis forest plot of apheresis session/patient by baseline IgG titer (≤ 64 vs > 64) to achieve pre‐KT IgG titer ≤ 16.

A meta‐analysis was conducted to compare the average number of apheresis sessions required per patient to achieve pre‐KT IgG titer ≤ 16, as most ABOi‐KT desensitization protocols require IgG titers to reach this level. Ten included studies [17, 19, 27, 29, 37, 41, 53, 55, 56, 59] were analyzed, comparing patients with baseline IgG titers ≤ 64 (101 patients) and > 64 (82 patients), as shown in Figure 6. Patients with baseline IgG titers ≤ 64 required a statistically significant lower average number of apheresis sessions per patient to reach the pre‐KT IgG titer ≤ 16 compared to those with baseline IgG titers > 64 (MD −1.07, 95% CI −1.4 to −0.74, *I* ^2^ 0%, *p* < 0.001). Similar results were observed in the PE subgroup meta‐analysis based on four included studies [29, 37, 53, 56] involving 91 patients (46 vs 45 patients) with statistically significant lower average PE sessions on patients with baseline IgG titers ≤ 64 as compared with patients with baseline IgG titers > 64 to reach the pre‐KT IgG titer ≤ 16 (MD −1.22, 95% CI −1.87 to −0.57, *I* ^2^ 27.67%, *p* < 0.001). Based on four included studies [17, 19, 27, 59] involving 58 patients (34 vs 24 patients), patients with baseline IgG titers ≤ 64 required a lower average number of IA sessions per patient to reach the pre‐KT IgG titer ≤ 16 compared to patients with baseline IgG titers > 64, although not statistically significant (MD −1.19, 95% CI −2.4 to 0.03, *I* ^2^ 75.36%, *p* = 0.05). The PE and IA combination subgroup meta‐analysis, based on two included studies [41, 55] involving 34 patients (21 vs. 13 patients), showed a statistically significant fewer sessions per patient in patients with baseline IgG ≤ 64 compared to > 64 to reach the pre‐KT IgG titer ≤ 16 (MD −1.04, 95% CI −1.74 to −0.33, *I* ^2^ 0%, *p* = 0.04). Subgroup meta‐analyses based on publication decade (2000–2009, 2010–2019, and 2020–2024) were also performed with two [17, 19], five [27, 29, 37, 41, 44], and four [53, 55, 56, 59] included studies involving 35 (19 vs 16 patients), 50 (27 vs 23 patients), 98 (55 vs 43 patients), respectively. Subgroup meta‐analysis of studies published between 2000 and 2009 showed a lower average number of apheresis sessions required per patient to achieve pre‐KT IgG titer ≤ 16 on patients with baseline IgG titers ≤ 64 as compared with patients with baseline IgG titers > 64, although not statistically significant (MD −0.71, 95% CI −1.41 to −0.01, *I* ^2^ 0%, *p* = 0.05). Similarly, subgroup meta‐analyses of studies published between 2010 and 2019 and studies published between 2020 and 2024 showed a statistically significant lower average number of apheresis sessions required per patient to achieve pre‐KT IgG titer ≤ 16 on patients with baseline IgG titers ≤ 64 as compared with patients with baseline IgG titers > 64 (MD −1.89, 95% CI −2.56 to −1.22, *I* ^2^ 0%, *p* < 0.001; MD −0.78, 95% CI −1.25 to −0.3, *I* ^2^ 0%, *p* = 0.001). Subgroup meta‐analyses of cohort studies with four included studies [37, 55, 56, 59] involving 88 patients (39 vs 49 patients) and case series studies with six included studies [17, 19, 27, 29, 41, 53] involving 95 (62 vs 33 patients) also showed a statistically significant lower average number of apheresis sessions required per patient to achieve pre‐KT IgG titer ≤ 16 on patients with baseline IgG titers ≤ 64 as compared with patients with baseline IgG titers > 64 (MD −1.23, 95% CI −1.83 to −0.63, *I* ^2^ 25.18%, *p* < 0.001; MD −0.94, 95% CI (−1.39 to −0.48, *I* ^2^ 0%, *p* < 0.001). However, as mentioned before, a potential study by Castro et al. [44] was excluded due to an unusually high number of PE sessions required per patient, which could have significantly altered the results. Overall, substantial heterogeneity was observed across analyses, with *I* ^2^ values ranging from 69.64% to 81.49%. Multivariable metaregression analyses showed a statistically significant higher apheresis session required per patient on PE as compared with IA or PE and IA combination as presented in Table 4. However, publication decade and study type showed no statistically significant association on apheresis sessions required per patient. An univariable metaregression analysis also showed no statistically significant associations between publication year and apheresis session required per patient as presented in Figure 7.

FIGURE 7Univariable metaregression analysis plots of (a) apheresis session/patient and (b) apheresis session/patient by baseline IgG titer (≤ 64 vs > 64) to achieve pre‐KT IgG titer ≤ 16.

**Figure:**
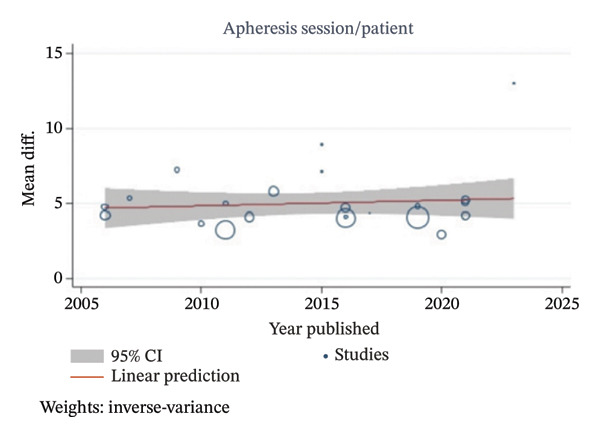
(a)

**Figure:**
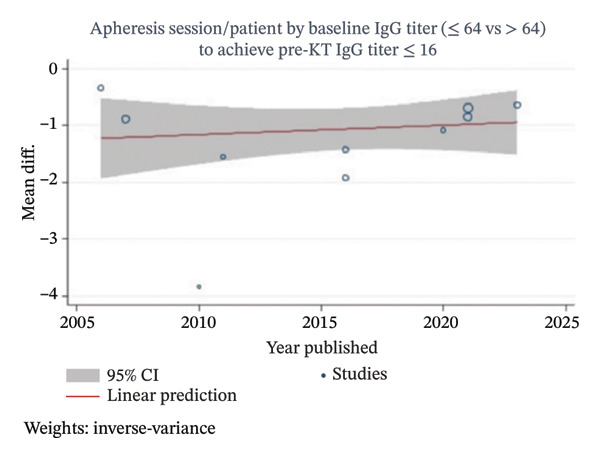
(b)

### 3.4. Risk of Bias Analysis and Quality Assessment

As shown in Supporting Table 3, six included cohort studies [21, 23, 28, 46, 52, 54] were categorized as “moderate” risk of bias, while nineteen included cohort studies [24, 25, 30, 33, 34, 36–39, 42, 45, 48, 49, 51, 55, 56, 58–60] were categorized as “low” risk of bias. The “moderate” risk of bias cohort studies had quality scores ranging from 54.55% to 63.64%, whereas the “low” risk of bias cohort studies had scores ranging from 72.73% to 90.91%. Potential biases were noted in the strategies used to address confounding factors and in determining whether participants were free of the outcome at the start of the study (or at the time of exposure).

Three included case series studies [16, 17, 47] were categorized as “moderate” risk of bias, with scores ranging from 50% to 60%. The remaining twenty included case series studies [13–15, 18–20, 22, 26, 27, 29, 31, 32, 35, 40, 41, 43, 44, 50, 53, 57] were categorized as “low” risk of bias. Majority of the potential biases in these studies were related to unclear criteria for patient inclusion, uncertainty regarding the completeness and consecutiveness of patient recruitment, and inadequate reporting of demographic information.

As shown in Table 3, publication bias was detected in the meta‐analyses of overall average number of apheresis sessions required per patient as reflected by Egger’s and Begg’s test results (*p* < 0.001 and *p* = 0.04). However, subgroup meta‐analyses of PE also showed a statistically significant Egger’s test (*p* = 0.006). Publication bias was also detected in the subgroup meta‐analyses for publication decades 2010–2019 and 2020–2024 and also in the study type subgroup meta‐analyses for cohort studies and case series studies, as reflected by statistically significant Egger’s test results (*p* = 0.03, *p* = 0.02, *p* = 0.04, and *p* = 0.003, respectively). Meta‐analysis of overall comparison of average number of apheresis sessions required per patient to achieve pre‐KT IgG titer ≤ 16 between baseline IgG titer ≤ 64 and > 64 also showed a potential publication bias as reflected by a statistically significant Egger’s test result (*p* = 0.04). This potential bias may be due to the following factors: (1) small sample sizes in several included studies, especially case series studies; (2) significant variability in desensitization methods and timing, including the agent of immunosuppression and additional splenectomy; (3) variations in apheresis methods and dosages, including PE types (e.g., DFFP or selective plasma exchange [SePE]) and IA types (e.g., semiselective IA, selective IA, and additional columns or reused columns); and (4) wide differences in baseline ABO allo‐isoagglutinin (IgG or IgM with anti‐A and/or anti‐B) titers, which may influence the number of required apheresis sessions. To mitigate potential publication bias, we performed subgroup meta‐analyses based on apheresis types, publication decade, and study types on each meta‐analysis to better understand the heterogeneity in study data. Statistically significant Egger’s test and/or Begg’s test on subgroup meta‐analyses outcomes may indicate the source for heterogeneity in the overall meta‐analyses. We also excluded one potential outlier study, Castro et al. [44], due to abnormally high apheresis sessions required per patient. Sensitivity analyses for all meta‐analyses showed that no single study significantly altered the overall results, as illustrated in Supporting Figures 1, 2, 3, and 4, supporting the robustness of the findings. Sensitivity analyses for all meta‐analyses with case series study exclusion (cohort only studies) also showed no single study significantly may alter the overall results, as presented in Supporting Figures 5, 6, 7, and 8.

## 4. Discussion

Apheresis, in combination with immunosuppressive agents, has become the cornerstone of the desensitization protocol in ABOi‐KT today [61]. Since the main barrier to ABOi‐KT is high ABO allo‐isoagglutinin IgG and/or IgM (anti‐A and/or anti‐B) titers, the primary rationale for using apheresis is to reduce these titers [3, 61]. Since the first large study by Alexandre et al. in 1985 that incorporated apheresis as part of the desensitization protocol, ABOi‐KT has become more feasible, offering the potential to expand the donor pool and reduce both morbidity and mortality [62]. However, apheresis‐based desensitization regimens have not been uniformly adopted and have evolved differently across centers, as reflected by the varied desensitization protocol in our included studies [13–60]. The effectiveness of apheresis in reducing ABO allo‐isoagglutinin IgG and/or IgM (anti‐A and/or anti‐B) titers remains a matter of debate. Moreover, there is no universal consensus on the optimal apheresis technique or the number of sessions required for desensitization in ABOi‐KT. Even the acceptable pre‐transplant titer level is not standardized; while the majority of protocols use 1:16 as the upper limit, others adopt 1:4 or 1:32 as thresholds [29, 63, 64]. This study was conducted to provide a broader and more detailed understanding of the effectiveness of various apheresis techniques and session requirements in reducing ABO allo‐isoagglutinin IgG and/or IgM (anti‐A and/or anti‐B) titers. The aim of this study is to be a consideration for clinical practice and the development of standardized apheresis‐based desensitization protocol guidelines for ABOi‐KT. To the best of the authors’ knowledge, this is the first comprehensive systematic review and meta‐analysis to evaluate the TRR of ABO allo‐isoagglutinin IgG and/or IgM (anti‐A and/or anti‐B) titers and the required number of apheresis sessions required per patient in the context of apheresis‐based desensitization for ABOi‐KT based on the current body of evidence.

The use of ABO allo‐isoagglutinin IgG or IgM (anti‐A and/or anti‐B) titer thresholds as a standard criterion for undergoing ABOi‐KT remains unclear due to the unique characteristics of these antibodies [63]. Some centers rely on IgG titers, while others use IgM titers, with insufficient evidence that high pre‐KT IgG titers can reliably predict AMR [54]. However, both IgG and IgM TRR in our results showed statistically significant reductions, with average decreases of 13.27‐fold and 14.12‐fold, respectively. This suggests that either IgG or IgM titer thresholds could potentially serve as viable standards for ABOi‐KT, as both are significantly reduced following apheresis‐based desensitization. Among the techniques evaluated, IA achieved the highest IgG TRR, with an average 17.27‐fold reduction, followed by PE and the combination of PE and IA, which showed 12.3‐fold and 12.9‐fold reductions, respectively. This may be attributed to the selective or semiselective nature of IA, which allows for more targeted removal of IgG compared to the broader action of PE [65]. Conversely, IA had the lowest IgM TRR, averaging an 11.63‐fold reduction, compared to 17.39‐fold and 11.96‐fold reductions for PE and the combination of PE and IA, respectively. This may be explained by the variation in IgM binding sites depending on the type of semiselective IA column used, as some semiselective columns exhibit limited IgM binding capacity [66, 67]. Incorporating additional membranes or specific types of IA columns may help address this limitation and reduce the need for additional apheresis sessions [65–67]. Selective IA may also serve as an effective option to achieve greater reductions in ABO allo‐isoagglutinin IgG and/or IgM (anti‐A and/or anti‐B) titers. However, the limited availability and cost of selective IA in some regions may hinder its widespread adoption.

In our study, the average number of apheresis sessions required per patient for desensitization in ABOi‐KT was statistically significant, with an average of 4.97x sessions per patient. Similar average numbers were observed in patients receiving IA and a combination of PE and IA, with means of 6.36x and 6.38x sessions per patient, respectively. A lower average was observed in patients with PE alone, at 4.32x sessions per patient. These findings differ from those reported in a previous meta‐analysis by Lo et al. in 2016 [68], which stated that an average of 4 apheresis sessions (using PE, IA, or both) was typically required for desensitization in ABOi‐KT, with additional sessions necessary if the baseline ABO allo‐isoagglutinin IgG and/or IgM (anti‐A and/or anti‐B) titer exceeded 1:256. We believe the discrepancies between our findings and those of Lo et al. may be attributed to differences in baseline ABO allo‐isoagglutinin IgG and/or IgM titers among patients undergoing different apheresis techniques.

Our results demonstrated that patients with baseline IgG titers ≤ 64 required fewer apheresis sessions to achieve a pre‐KT IgG titer of ≤ 16, with an average difference of 1.65x sessions per patient compared to those with IgG titers > 64. Similar patterns were observed within IA and PE subgroups, with average session differences of 1.56x and 1.64x sessions required per patient, respectively. These findings suggest that a difference of 1‐2 apheresis sessions may be sufficient to achieve a safe titer threshold for ABOi‐KT, with the commonly accepted upper limit of 1:16 for pre‐KT IgG and/or IgM titers [64].

In ABOi‐KT, isoagglutinins are directly implicated in AMR pathogenesis, and laboratory reviews emphasize that titer monitoring and reduction below a critical threshold are central components of risk control [69]. In this context, our IgG and IgM TRR findings provide clinically useful evidence that apheresis can achieve substantial antibody reduction across modalities, while our session per patient results add practical information on treatment intensity and resource burden needed to reach transplant readiness [65]. Importantly, contemporary outcome studies and reviews show that ABOi‐KT can achieve favorable graft and patient survival when desensitization protocols successfully control isoagglutinin titers, including selected patients with high baseline titers, although AMR and infectious complications remain important risks that require careful protocol selection and monitoring [65]. Therefore, our results strengthen the clinical utility of IgG and IgM TRR and apheresis session metrics as operational markers of desensitization effectiveness, while also supporting the need for future studies to report these metrics alongside target titer attainment, rebound, AMR, and graft survival to better define which apheresis strategy provides the best overall transplant outcomes [69].

Apheresis‐based desensitization methods in ABOi‐KT should take into account both patient‐specific factors and the characteristics of the apheresis technique to ensure a safe transplant. The appropriate choice of apheresis technique should be guided by the type of concurrent immunosuppressive therapy, the patient’s hemodynamic condition, and the availability of the apheresis method at the treatment center. Additionally, the TRR of IgG and IgM, as well as the number of apheresis sessions required per patient, can serve as important considerations when selecting the most suitable apheresis technique.

### 4.1. Limitation

This study has several limitations. First, the included studies consisted of prospective and retrospective cohort studies, as well as case series with a limited number of patients due to the lack of available RCTs that fulfill inclusion criteria. This may introduce potential selection bias and limit the generalizability of the findings to a broader population. Second, the baseline characteristics of the included studies varied considerably, particularly regarding ABO allo‐isoagglutinin IgG and/or IgM (anti‐A and/or anti‐B) titers. The difference in laboratory methods and target titer threshold also may contribute to the high heterogeneity. This may contribute to the varied IgG and IgM TRR and also the apheresis sessions required per patient. Third, the apheresis techniques used in desensitization differed widely, ranging from conventional PE, selective PE (SePE), and DFPP to selective and semiselective IA with different columns used. These factors likely contributed to substantial heterogeneity in our analyses. Fourth, publication bias was detected in the analyses of apheresis sessions required per patient and apheresis sessions required per patient comparison stratified by baseline IgG titer (≤ 64 vs > 64) to achieve a pre‐KT IgG titer ≤ 16. However, sensitivity analyses for all of the meta‐analyses demonstrated that the results remained consistent. Sensitivity analyses based on cohort studies were conducted in all outcomes with also showing no alteration in the result indicating robust results. Fifth, metaregression was limited to study‐level covariates with sufficiently consistent reporting (apheresis type, publication decade, and study type). Sixth, a formal pooled secondary analysis of AMR and graft survival in relation to IgG and IgM TRR was not feasible because outcome definitions and reporting were not sufficiently standardized across included studies. Variables such as rituximab use, IA column subtype, and protocol details could not be metaregressed reliably due to incomplete and not detailed reporting across studies. Seventh, the publication years of the included studies spanned from 2000 to 2023, during which apheresis techniques and their modifications have evolved substantially. This temporal variability may limit the applicability of our findings to current clinical practice. Despite these limitations, this study offers the most up‐to‐date evidence on the effectiveness of apheresis in reducing ABO allo‐isoagglutinin IgG and/or IgM (anti‐A and/or anti‐B) titers in ABOi‐KT.

## 5. Conclusion

This systematic review and meta‐analysis suggest that apheresis significantly reduces ABO allo‐isoagglutinin IgG and/or IgM (anti‐A and/or anti‐B) titers, with an approximate 13.5‐fold and 14.2‐fold reduction in IgG and IgM titer levels, respectively. On average, five apheresis sessions were required per patient, with a difference of approximately 1.5x sessions between patients with baseline IgG titers ≤ 64 and > 64 to achieve a pre‐KT IgG titer of ≤ 16. Although the apheresis methods and desensitization protocols varied across studies, including the use of PE, IA, and their combinations, our findings highlight the essential role of apheresis‐based desensitization in enabling safe and effective ABOi‐KT. The observed heterogeneity in apheresis techniques, IgG/IgM titer thresholds, and session requirements underscores the need for standardized desensitization protocols. Future large‐scale RCTs are warranted to develop evidence‐based guidelines and optimize apheresis‐based desensitization strategies in ABOi‐KT.

## Funding

No funding was received for this manuscript.

## Conflicts of Interest

The authors declare no conflicts of interest.

## Supporting Information

Supporting Table 1: keywords used in literature search.

Supporting Table 2: baseline characteristics of included studies.

Supporting Table 3: risk of bias analysis with JBI critical appraisal tools.

Supporting Figure 1: sensitivity analysis forest plot of IgG TRR.

Supporting Figure 2: sensitivity analysis forest plot of IgM TRR.

Supporting Figure 3: sensitivity analysis forest plot of apheresis session/patient.

Supporting Figure 4: sensitivity analysis forest plot of apheresis session/patient by baseline IgG titer (≤ 64 vs > 64) to achieve pre‐KT IgG titer ≤ 16.

Supporting Figure 5: cohort study sensitivity analysis forest plot of IgG TRR.

Supporting Figure 6: cohort study sensitivity analysis forest plot of IgM TRR.

Supporting Figure 7: cohort study sensitivity analysis forest plot of apheresis session/patient.

Supporting Figure 8: cohort study sensitivity analysis forest plot of apheresis session/patient by baseline IgG titer (≤ 64 vs > 64) to achieve pre‐KT IgG titer ≤ 16.

## Supporting information


**Supporting Information** Additional supporting information can be found online in the Supporting Information section.

## Data Availability

The data that support the findings of this study are available on request from the corresponding author. The data are not publicly available due to privacy or ethical restrictions.
