# Supplementary material for: Apheresis‐Based Desensitization to Reduce Antibody Titer in ABO‐Incompatible Kidney Transplantation: A Systematic Review and Meta‐Analysis
Source: J Transplant. 2026 Apr 29;2026:5848415. doi: 10.1155/joot/5848415 (PMC13126248; doi:10.1155/joot/5848415)
Supplement: Supplementary file 1 — Supporting Information Additional supporting information can be found online in the Supporting Information section. [file JOOT-2026-5848415-s001.docx]

Supplementary Materials

#### Supplementary Table 1. Keywords used in literature search.

| Database | Keywords | | Results |
| --- | --- | --- | --- |
| Pubmed | #1 | ("blood group incompatibility"[MeSH Terms]) OR ("abo-incompatible"[Title/Abstract]) OR ("abo incompatible"[Title/Abstract]) OR ("abo-i"[Title/Abstract]) OR ("aboi"[Title/Abstract]) | 9442 |
|  | #2 | ("kidney transplantation"[MeSH Terms]) OR ("kidney transplant*"[Title/Abstract]) OR ("kidney-transplant*"[Title/Abstract]) OR ("renal transplant*"[Title/Abstract]) OR ("renal-transplant*"[Title/Abstract]) | 132780 |
|  | #3 | ("blood component removal"[MeSH Terms]) OR ("plasmapheresis"[MeSH Terms]) OR ("plasma exchange"[MeSH Terms]) OR ("apheresis"[Title/Abstract]) OR ("hemapheresis"[Title/Abstract]) OR ("pheresis"[Title/Abstract]) OR ("plasmapheresis"[Title/Abstract]) OR ("plasma exchange"[Title/Abstract]) OR ("immunoadsorption"[Title/Abstract]) OR ("immunoapheresis"[Title/Abstract]) OR ("titer*"[Title/Abstract]) | 152241 |
|  | #4 | (#1 AND #2) AND #3 | 547 |
| CENTRAL | #1 | MeSH descriptor: [Blood Group Incompatibility] explode all trees | 98 |
|  | #2 | (abo-incompatible OR abo incompatible OR abo-i OR aboi):ti,ab,kw | 109 |
|  | #3 | #1 OR #2 | 187 |
|  | #4 | MeSH descriptor: [Kidney Transplantation] explode all trees | 4664 |
|  | #5 | (kidney transplant* OR kidney-transplant* OR renal transplant* OR renal-transplant*):ti,ab,kw | 15847 |
|  | #6 | #4 OR #5 | 15847 |
|  | #7 | #3 AND #6 | 54 |
|  | #8 | MeSH descriptor: [Blood Component Removal] explode all trees | 1242 |
|  | #9 | MeSH descriptor: [Plasmapheresis] explode all trees | 552 |
|  | #10 | MeSH descriptor: [Plasma Exchange] explode all trees | 326 |
|  | #11 | (apheresis OR hemapheresis OR pheresis OR plasmapheresis OR plasma exchange OR immunoadsorption OR immunoapheresis OR titer*):ti,ab,kw | 12390 |
|  | #12 | #8 OR #9 OR #10 OR #11 | 12766 |
|  | #13 | #7 AND #12 | 23 |
| Scopus | TITLE-ABS-KEY ( "abo-incompatible" OR "abo incompatible" OR "abo-i" OR "aboi" ) AND TITLE-ABS-KEY ( "kidney transplant*" OR "kidney-transplant*" OR "renal transplant*" OR "renal-transplant*" ) AND TITLE-ABS-KEY ( "apheresis" OR "hemapheresis" OR "pheresis" OR "plasmapheresis" OR "plasma exchange" OR "immunoadsorption" OR "immunoapheresis" OR "titer*" ) | | 729 |
| ProQuest | noft(abo-incompatible OR abo incompatible OR abo-i OR aboi) AND noft(kidney transplant* OR kidney-transplant* OR renal transplant* OR renal-transplant*) AND noft(apheresis OR hemapheresis OR pheresis OR plasmapheresis OR plasma exchange OR immunoadsorption OR immunoapheresis OR titer*) | | 50 |
| ScienceDirect | ( "abo-incompatible" OR "abo incompatible") AND ( "kidney transplant"") AND ("plasmapheresis" OR "plasma exchange" OR "immunoadsorption" OR "titer" ) | | 133 |
| MEDLINE | #1 | MHX=(blood group incompatibility) OR TI=(abo-incompatible OR abo incompatible OR abo-i OR aboi) OR AB=(abo-incompatible OR abo incompatible OR abo-i OR aboi) | 6513 |
|  | #2 | MHX=(kidney transplantation) OR TI=(kidney transplant* OR kidney-transplant* OR renal transplant* OR renal-transplant*) OR AB=(kidney transplant* OR kidney-transplant* OR renal transplant* OR renal-transplant*) | 156247 |
|  | #3 | #1 AND #2 | 1463 |
|  | #4 | MHX=(blood component removal) OR TI=(plasmapheresis OR plasma exchange OR apheresis OR hemapheresis OR pheresis OR plasmapheresis OR plasma exchange OR immunoadsorption OR immunoapheresis OR titer*) OR AB=(plasmapheresis OR plasma exchange OR apheresis OR hemapheresis OR pheresis OR plasmapheresis OR plasma exchange OR immunoadsorption OR immunoapheresis OR titer*) | 161717 |
|  | #5 | #3 AND #4 | 561 |

#### Supplementary Table 2. Baseline characteristics of included studies.

| Study | Study type | Study period | Study origin | Patients | | | Apheresis | | |
| --- | --- | --- | --- | --- | --- | --- | --- | --- | --- |
|  |  |  |  | n | M/F | Age (yr) | Type | No. apheresis sessions | Apheresis session/patient |
| Ohta et al 2000 [13] | CS | 1989-1997 | Japan | 10 | 6/4 | 13.45^a^±3.37^b^ | PE, IA | NI | NI |
| Shishido et al 2001 [14] | CS | 1989-2000 | Japan | 16 | 13/3 | 10.34^a^±3.16^b^ | PE, IA | NI | NI |
| Tydén et al 2005 [15] | CS | Since 2001* | Sweden | 10 | 5/5 | 35.3^a^±16.15^b^ | IA | 49 | 4.9^a^±1.91^b^ |
| Kumlien et al 2006 [16] | CS | Since 2001* | Sweden | 13 | NI | NI | IA | 65 | 5^a^±1.91^b^ |
| Nordén et al 2006 [17] | CS | Since 2002* | Sweden | 14 | NI | 47.86^a^±12.94^b^ | IA | 59 | 4.21^a^±0.97^b^ |
| Tydén et al 2006 [18] | CS | Since 2001* | Sweden | 19 | NI | NI | IA | 90 | 4.74^a^±1.63^b^ |
| Wilpert et al 2007 [19] | CS | Since 2004* | Germany | 22 | 13/9 | 44.95^a^±10.75^b^ | IA | 117 | 5.32^a^±2.61^b^ |
| Ignjatović et al 2009 [20] | CS | 2006-2008 | Serbia | 12 | 6/6 | 40.33^a^±7.52^b^ | PE, IA | 87 | 7.25^a^±1.66^b^ |
| Montgomery et al 2009 [21] | RC | 1999-2007 | USA | 60 | 29/31 | 50.3^a^±11.46^b^ | PE | NI | 7.64^a^±6.27^b^ |
| Sivakumaran et al 2009 [22] | CS | 2006-2008 | USA | 10 | 5/5 | 44.7^a^±14.3^b^ | PE | 50 | 5^a^±0^b^ |
| Tobian et al 2009 [23] | RC | 1999-2007 | USA | 46 | NI | NI | PE | NI | 6.2^a^±2.5^b^ |
| Toki et al 2009_1 [24] | RC | 1999-2004 | Japan | 22 | 12/10 | 38.91^a^±12.63^b^ | PE | NI | NI |
| Toki et al 2009_2 [25] | RC | 1990-2007 | Japan | 14 | 8/6 | 42.29^a^±11.1^b^ | PE | NI | NI |
| Valli et al 2009 [26] | CS | 2004-2007 | Germany, Switzerland | 17 | 13/4 | 46.88^a^±9.66^b^ | IA | 102 | 6^a^±3.61^b^ |
| Schousboe et al 2010 [27] | CS | Since 2007* | Denmark | 11 | 10/1 | 49.73^a^±13.08^b^ | IA | 40 | 3.64^a^±1.43^b^ |
| Tobian et al 2010 [28] | RC | 1999-2007 | USA | 13 | 5/8 | NI | PE | NI | NI |
| Chung et al 2011 [29] | CS | 2009-2010 | South Korea | 14 | 9/5 | 45.43^a^±9.8^b^ | PE | 120 | 8.57^a^±3.55^b^ |
| Shirakawa et al 2011 [30] | RC | 2005-2010 | Japan | 74 | 52/22 | 46.24^a^±14.25^b^ | PE | NI | 3.22^a^±1.16^b^ |
| Uchida et al 2011 [31] | CS | 1999-2007 | Japan | 12 | 7/5 | 55.17^a^±4.99^b^ | PE | 60 | 5^a^±2^b^ |
| Yoo et al 2012 [32] | CS | 2010-2011 | South Korea | 12 | 5/7 | 48.75^a^±9.85^b^ | PE | 49 | 4.08^a^±0.9^b^ |
| Kong et al 2013 [33] | RC | 2007-2010 | South Korea | 118 | 76/42 | 44^a^±11^b^ | PE | NI | 5.8^a^±2.7^b^ |
| Won et al 2014 [34] | RC | 2009-2012 | South Korea | 95 | 65/30 | 44.6^a^±12.1^b^ | PE | NI | 4.2^a^±1.5^b^ |
| Maggioni et al 2015 [35] | CS | Since 2011* | France | 19 | 10/9 | 44.53^a^±14.6^b^ | PE, IA | 136 | 7.16^a^±4.63^b^ |
| Thölking et al 2015 [36] | RC | 2009-2014 | Germany | 23 | 15/8 | 44.04^a^±14.34^b^ | IA | NI | 8.93^a^±6.66^b^ |
| Jha et al 2016 [37] | RC | 2011-2014 | India | 17 | NI | NI | PE | 70 | 4.12^a^±3.16^b^ |
| Lee et al 2016 [38] | RC | 2010-2015 | South Korea | 59 | 37/22 | 46^a^±10.4^b^ | PE | NI | 4.72^a^±2.15^b^ |
| Ray and Thukral 2016 [39] | RC | 2013-2015 | India | 45 | 36/9 | 43^a^±12.5^b^ | PE | NI | 4^a^±0.9^b^ |
| Rostaing et al 2016_1 [40] | CS | NI | France | 12 | 13/7 | 42^a^±12.49^b^ | IA | NI | NI |
| Rostaing et al 2016_2 [41] | CS | Since 2011* | France | 12 | 5/7 | 45.25^a^±15.96^b^ | PE, IA | 162 | 13.5^a^±5.32^b^ |
| Sánchez-Escuredo et al 2016 [42] | PC | 2009-2013 | Spain | 30 | 20/10 | 44^a^±13^b^ | PE, IA | NI | IA: 6^c^ (5-12^d^), PE: 7^c^ (2-15^d^) |
| Tan et al 2016 [43] | CS | 2011-2013 | Malaysia | 10 | NI | 41.6^a^±8.22^b^ | PE, IA | 44 | 4.4^a^±1.58^b^ |
| Castro et al 2017 [44] | CS | 2012-2016 | Brazil | 10 | NI | 45.7^a^±10.49^b^ | PE | 422 | 42.2^a^±18.2^b^ |
| Lonze et al 2017 [45] | RC | 1999-2012 | USA | 115 | 65/50 | 47^a^±13^b^ | PE | NI | 4.35^a^±2.25^b^ |
| Makroo et al 2017 [46] | PC | 2012-2015 | India | 29 | 22/7 | 38.7^a^ (17-75^d^) | PE | 139 | 4.8^a^ |
| Shah et al 2017 [47] | CS | NI | India | 19 | NI | NI | PE, IA | NI | NI |
| Agrawal et al 2019 [48] | RC | PE: 2012-2015, IA±PE: 2015-2017 | India | PE: 29, IA±PE: 11 | 32/8 | 36.9^a^±14.51^b^ | PE, IA | NI | PE (4.8^a^±2.5^b^), IA±PE (PE: 3.5^a^±2.4^b^, IA: 1.6^a^±0.5^b^) |
| Baek et al 2019 [49] | RC | 2009-2013 | South Korea | 180 | 120/60 | 46.34^a^±11.51^b^ | PE | NI | 4.08^a^±1.51^b^ |
| Hanaoka et al 2019 [50] | CS | 2015-2017 | Japan | 15 | 9/6 | 51.4^a^±10.84^b^ | PE | 53 | 3.53^a^±2.97^b^ |
| Speer et al 2019 [51] | RC | 2005-2018 | Germany | 48 | 28/20 | 44.37^a^±10.55^b^ | PE, IA | NI | IA: 6^c^ (2-13^d^), PE: 2^c^ (2-6^d^) |
| Thukral et al 2019 [52] | PC | 2014-2015 | India | 30 | NI | NI | PE | NI | 3-7^d^ |
| Hanaoka et al 2020 [53] | CS | 2015-2018 | Japan | 30 | 22/8 | 52.93^a^±12.71^b^ | PE | 88 | 2.93^a^±1.55^b^ |
| Kim et al 2020 [54] | RC | 2012-2014 | South Korea | 120 | 78/42 | 47.29^a^±10.33^b^ | PE | NI | 3.69^a^±1.32^b^ |
| Gan et al 2021 [55] | RC | 2008-2018 | Malaysia | 25 | 14/11 | 39.84^a^±11.34^b^ | PE, IA | 127 | 5.08^a^±1.87^b^ |
| Pandey et al 2021_1 [56] | RC | 2015-2018 | India | 36 | 27/9 | 42.36^a^±12.53^b^ | PE | 150 | 4.17^a^±1.83^b^ |
| Pandey et al 2021_2 [57] | CS | 2015-2018 | India | 10 | 5/5 | 47.1^a^±12.97^b^ | PE | 54 | 5.4^a^±1.65^b^ |
| Yachha et al 2021 [58] | RC | 2013-2016 | India | 33 | 31/2 | 35^a^±11.07^b^ | PE | NI | 5.23^a^±1.92^b^ |
| Junker et al 2023 [59] | RC | 2013-2019 | Switzerland | 13 | 10/3 | 51.05^a^±13.75^b^ | IA | 69 | 5.31^a^±1.65^b^ |
| Okada et al 2023 [60] | RC | 2007-2021 | Japan | 142 | 100/42 | 53.01^a^±11.6^b^ | PE | NI | NI |

No = amount, PC = prospective cohort; RC = retrospective cohort; CS = case series; PE = plasma exchange; IA = Immunoadsorption; NI = not information; * = not detailed in the study; M = male; F = Female; n = amount; ^a^ = mean; ^b^ = standard deviation; ^c^ = median; ^d^ = range

#

#

#

#

#

#

#

#

#

#

#

#

#

#

#

#

#

#

#

#

#

#

#

#

#

#

#

#

####

#### Supplementary Table 3. Risk of bias analysis with JBI critical appraisal tools.

| Study | Q1 | Q2 | Q3 | Q4 | Q5 | Q6 | Q7 | Q8 | Q9 | Q10 | Q11 | Total (%) | Overall |
| --- | --- | --- | --- | --- | --- | --- | --- | --- | --- | --- | --- | --- | --- |
| Cohort studies | | | | | | | | | | | | | |
| Montgomery et al 2009 [21] | Y | Y | Y | U | U | U | Y | Y | Y | U | Y | 7 (63.64) | Moderate |
| Tobian et al 2009 [23] | Y | Y | Y | U | U | U | Y | Y | Y | U | Y | 7 (63.64) | Moderate |
| Toki et al 2009_1 [24] | Y | Y | Y | U | U | Y | Y | Y | Y | U | Y | 8 (72.73) | Low |
| Toki et al 2009_2 [25] | Y | Y | Y | U | U | Y | Y | Y | Y | U | Y | 8 (72.73) | Low |
| Tobian et al 2010 [28] | Y | Y | Y | U | U | U | Y | Y | Y | U | Y | 7 (63.64) | Moderate |
| Shirakawa et al 2011 [30] | Y | Y | Y | U | U | Y | Y | Y | Y | U | Y | 8 (72.73) | Low |
| Kong et al 2013 [33] | Y | Y | Y | U | U | Y | Y | Y | Y | U | Y | 8 (72.73) | Low |
| Won et al 2014 [34] | Y | Y | Y | U | U | Y | Y | Y | Y | U | Y | 8 (72.73) | Low |
| Thölking et al 2015 [36] | Y | Y | Y | Y | U | Y | Y | Y | Y | U | Y | 9 (81.82) | Low |
| Jha et al 2016 [37] | Y | Y | Y | Y | U | Y | Y | Y | Y | U | Y | 9 (81.82) | Low |
| Lee et al 2016 [38] | Y | Y | Y | Y | U | Y | Y | Y | Y | U | Y | 9 (81.82) | Low |
| Ray and Thukral 2016 [39] | Y | Y | Y | Y | U | U | Y | Y | Y | U | Y | 8 (72.73) | Low |
| Sánchez-Escuredo et al 2016 [42] | Y | Y | Y | Y | U | U | Y | Y | Y | U | Y | 8 (72.73) | Low |
| Lonze et al 2017 [45] | Y | Y | Y | Y | U | Y | Y | Y | Y | Y | Y | 10 (90.91) | Low |
| Makroo et al 2017 [46] | U | Y | Y | U | U | Y | Y | Y | U | U | Y | 6 (54.55) | Moderate |
| Agrawal et al 2019 [48] | Y | Y | Y | Y | U | U | Y | Y | Y | U | Y | 8 (72.73) | Low |
| Baek et al 2019 [49] | Y | Y | Y | Y | Y | Y | Y | Y | Y | U | Y | 10 (90.91) | Low |
| Speer et al 2019 [51] | Y | Y | Y | Y | Y | Y | Y | Y | Y | U | Y | 10 (90.91) | Low |
| Thukral et al 2019 [52] | Y | Y | Y | Y | U | U | Y | Y | U | U | Y | 7 (63.64) | Moderate |
| Kim et al 2020 [54] | Y | Y | Y | U | U | U | Y | Y | Y | U | Y | 7 (63.64) | Moderate |
| Gan et al 2021 [55] | Y | Y | Y | Y | U | Y | Y | Y | Y | U | Y | 9 (81.82) | Low |
| Pandey et al 2021_1 [56] | Y | Y | Y | U | U | Y | Y | Y | Y | U | Y | 8 (72.73) | Low |
| Yachha et al 2021 [58] | Y | Y | Y | Y | Y | Y | Y | Y | Y | U | Y | 10 (90.91) | Low |
| Junker et al 2023 [59] | Y | Y | Y | Y | Y | Y | Y | Y | Y | U | Y | 10 (90.91) | Low |
| Okada et al 2023 [60] | Y | Y | Y | Y | Y | Y | Y | Y | Y | U | Y | 10 (90.91) | Low |
| Case series studies | | | | | | | | | | | | | |
| Ohta et al 2000 [13] | U | Y | Y | N | Y | Y | Y | Y | Y | N |  | 7 (70) | Low |
| Shishido et al 2001 [14] | Y | Y | Y | N | Y | Y | Y | Y | Y | N |  | 8 (80) | Low |
| Tydén et al 2005 [15] | U | Y | Y | N | Y | Y | Y | Y | Y | N |  | 7 (70) | Low |
| Kumlien et al 2006 [16] | U | Y | Y | N | Y | N | Y | Y | Y | N |  | 6 (60) | Moderate |
| Nordén et al 2006 [17] | Y | Y | Y | N | Y | U | Y | Y | U | N |  | 6 (60) | Moderate |
| Tydén et al 2006 [18] | Y | Y | Y | N | Y | N | Y | Y | Y | N |  | 7 (70) | Low |
| Wilpert et al 2007 [19] | Y | Y | Y | N | Y | Y | Y | Y | Y | Y |  | 9 (90) | Low |
| Ignjatović et al 2009 [20] | Y | Y | Y | N | Y | Y | Y | Y | Y | N |  | 8 (80) | Low |
| Sivakumaran et al 2009 [22] | Y | Y | Y | N | Y | Y | Y | Y | Y | N |  | 8 (80) | Low |
| Valli et al 2009 [26] | Y | Y | Y | N | Y | Y | Y | Y | Y | Y |  | 9 (90) | Low |
| Schousboe et al 2010 [27] | Y | Y | Y | N | Y | Y | Y | Y | Y | N |  | 8 (80) | Low |
| Chung et al 2011 [29] | Y | Y | Y | N | Y | Y | Y | Y | Y | N |  | 8 (80) | Low |
| Uchida et al 2011 [31] | Y | Y | Y | N | Y | Y | Y | Y | Y | N |  | 8 (80) | Low |
| Yoo et al 2012 [32] | Y | Y | Y | N | Y | Y | Y | Y | Y | N |  | 8 (80) | Low |
| Maggioni et al 2015 [35] | Y | Y | Y | N | Y | Y | Y | Y | U | N |  | 7 (70) | Low |
| Rostaing et al 2016_1 [40] | Y | Y | Y | Y | Y | Y | Y | Y | N | N |  | 8 (80) | Low |
| Rostaing et al 2016_2 [41] | Y | Y | Y | Y | Y | Y | Y | Y | N | N |  | 8 (80) | Low |
| Tan et al 2016 [43] | Y | Y | Y | Y | Y | Y | Y | Y | Y | N |  | 9 (90) | Low |
| Castro et al 2017 [44] | Y | Y | Y | U | Y | Y | Y | Y | Y | N |  | 8 (80) | Low |
| Shah et al 2017 [47] | Y | Y | Y | N | Y | U | U | Y | N | N |  | 5 (50) | Moderate |
| Hanaoka et al 2019 [50] | Y | Y | Y | Y | Y | Y | Y | Y | Y | N |  | 9 (90) | Low |
| Hanaoka et al 2020 [53] | Y | Y | Y | Y | Y | Y | Y | Y | Y | N |  | 9 (90) | Low |
| Pandey et al 2021_2 [57] | Y | Y | Y | Y | Y | Y | Y | Y | Y | N |  | 9 (90) | Low |

Q = question, Y = yes, U = unclear, N = no

#### Supplementary Figure 1. Sensitivity analysis forest plot of IgG TRR.


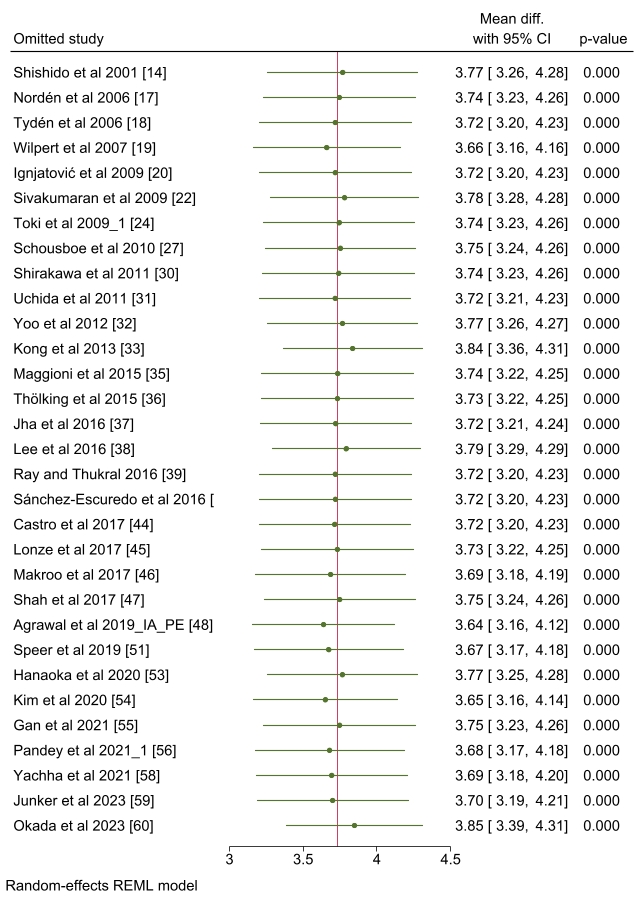


#### Supplementary Figure 2. Sensitivity analysis forest plot of IgM TRR.


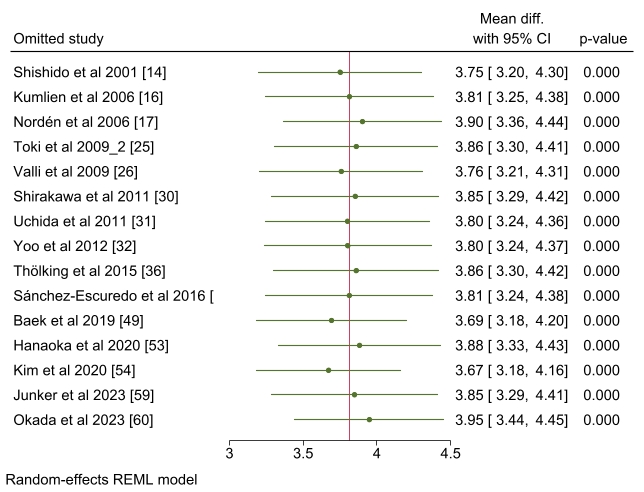


#### Supplementary Figure 3. Sensitivity analysis forest plot of apheresis session/patient.


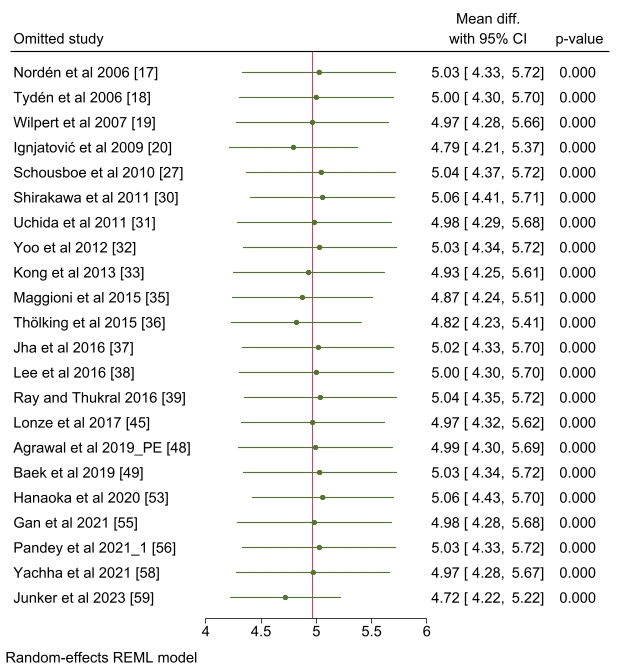


#### Supplementary Figure 4. Sensitivity analysis forest plot of apheresis session/patient by baseline IgG titer (≤64 vs >64) to achieve pre-KT IgG titer ≤16.


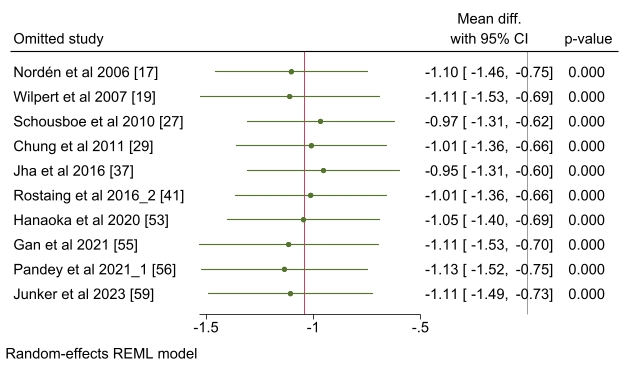


#### Supplementary Figure 5. Cohort studies sensitivity analysis forest plot of IgG TRR.


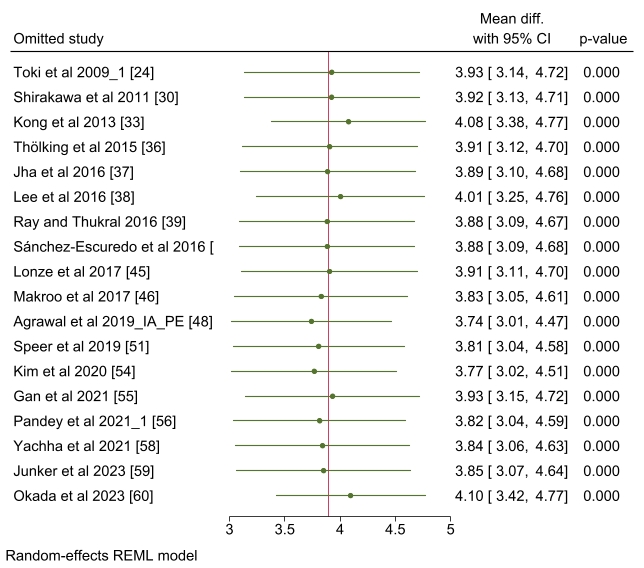


#### Supplementary Figure 6. Cohort studies sensitivity analysis forest plot of IgM TRR.


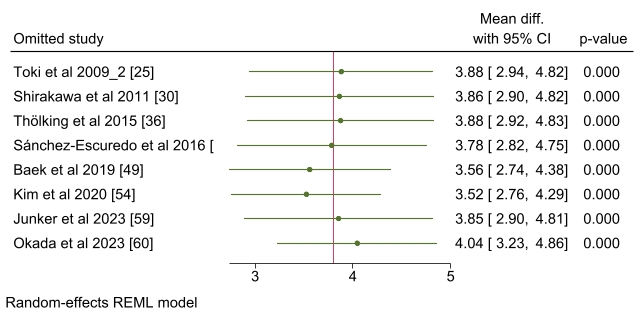


#### Supplementary Figure 7. Cohort studies sensitivity analysis forest plot of apheresis session/patient.


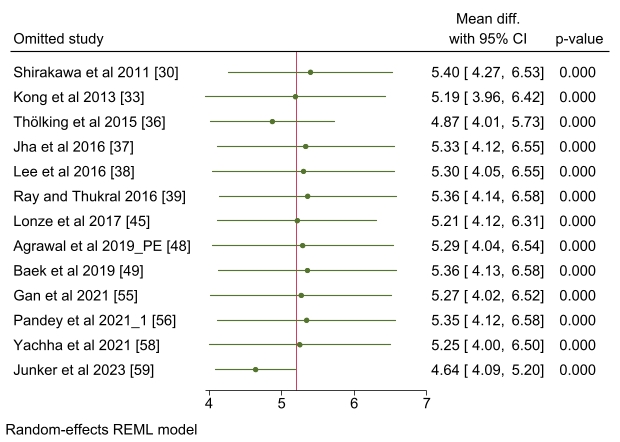


#### Supplementary Figure 8. Cohort studies sensitivity analysis forest plot of apheresis session/patient by baseline IgG titer (≤64 vs >64) to achieve pre-KT IgG titer ≤16.


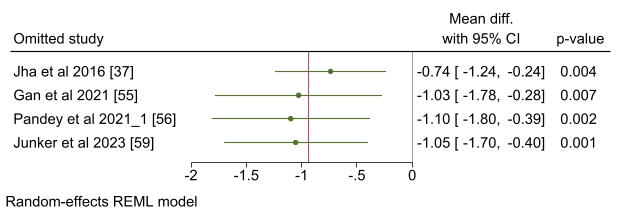


**Search Strategy**

We will perform our literature search based on a combination of MESH terms and keywords mentioned below. Preferred Reporting Items for Systematic Reviews and Meta-Analyses 2020 (PRISMA 2020 Statement) will be used as the guideline for literature search. Literature search will be systematically performed in 6 databases, PubMed, Cochrane Central Register of Controlled Trials (CENTRAL), Scopus, ProQuest, ScienceDirect, and MEDLINE

| Population | Type | Exposure | Type | Outcome | Type | Study Design | Type |
| --- | --- | --- | --- | --- | --- | --- | --- |
| blood group incompatibility | MESH | blood component removal | MESH | titer* | Title/Abstract | randomized controlled trial | MESH |
| abo-incompatible | Title/Abstract | plasmapheresis | MESH | Igg | Title/Abstract | randomized controlled trial | Title/Abstract |
| abo incompatible | Title/Abstract | plasma exchange | MESH | Igm | Title/Abstract | RCT | Title/Abstract |
| abo-i | Title/Abstract | apheresis | Title/Abstract | anti-a | Title/Abstract | observational study | MESH |
| aboi | Title/Abstract | hemapheresis | Title/Abstract | anti-b | Title/Abstract | observational study | Title/Abstract |
| kidney transplantation | MESH | pheresis | Title/Abstract |  |  | cohort study | Title/Abstract |
| kidney transplant* | Title/Abstract | plasmapheresis | Title/Abstract |  |  | case series | Title/Abstract |
| kidney-transplant* | Title/Abstract | plasma exchange | Title/Abstract |  |  |  |  |
| renal transplant* | Title/Abstract | immunoadsorption | Title/Abstract |  |  |  |  |
| renal-transplant* | Title/Abstract | immunoapheresis | Title/Abstract |  |  |  |  |
